# Fungal recognition in vaginal discharge using deep learning analysis of mobile device-acquired microscopic images

**DOI:** 10.3389/fcimb.2026.1787545

**Published:** 2026-03-12

**Authors:** Monsicha Pongpom, Siriwoot Sookkhee, Siriporn Chongkae, Sara Wattanasombat, Kornprom Pikulkaew, Narin Lawan, Phit Upaphong, Tanaporn Wangsanut

**Affiliations:** 1 Department of Microbiology, Faculty of Medicine, Chiang Mai University, Chiang Mai, Thailand; 2 Department of Computer Science, Faculty of Science, Chiang Mai University, Chiang Mai, Thailand; 3 Department of Chemistry, Faculty of Science, Chiang Mai University, Chiang Mai, Thailand; 4 Department of Ophthalmology, Faculty of Medicine, Chiang Mai University, Chiang Mai, Thailand

**Keywords:** Artificial intelligence, *Candida*, digital health, fungal image recognition, medical image analysis, mobile device, smartphone AI, vulvovaginal candidiasis

## Abstract

**Background:**

Vulvovaginal candidiasis (VVC) is a common fungal infection that is frequently diagnosed through manual microscopic examination of vaginal discharge. Artificial Intelligence (AI)-assisted analysis of microscopic images enables rapid and accurate diagnosis, supporting timely and effective antifungal therapeutic interventions. However, conventional light microscopy often lacks cameras, limiting digital image analysis and AI applications. While mobile devices offer a practical alternative, no AI tools currently exist for the automated detection of fungal cellular morphology in microscopic images captured by smartphones and tablets. In this study, we developed deep learning models to segment fungal morphologies in microscopic images of vaginal discharge acquired with smartphones and tablets.

**Methods:**

Three models were developed: ResNet18 for binary classification (*Candida* presence/absence), YOLOv5 for detection, and YOLOv11 for segmentation. Models were trained using 1,259 microscopy images of Gram-stained vaginal discharge acquired with smartphones or tablets, along with 67 images obtained from conventional microscopes. These images were divided into training, validation, and test sets. Annotated microscopic images for fungal elements were used to train YOLO models in a two-stage approach: Stage 1 utilized 687 annotated images of yeast infections to learn general fungal morphology, comprising 266 bounding box–annotated images sourced from Roboflow and 421 segmentation-labeled images manually annotated from the open-access dataset. Stage 2 fine-tuned the models on the annotated mobile device-acquired dataset. Metrics included F1-score, area under the curve (AUC), precision, recall, and mean average precision at 50% intersection over union (mAP50). Experts assessed segmentation outputs for diagnostic utility, providing explainability to the AI results.

**Results:**

ResNet18 achieved F1-score=0.986, AUC = 0.99. YOLOv5 performed best at IoU=0.50 (precision=0.812, recall=0.622, mAP50 = 0.730); YOLOv11 at IoU=0.25 (precision=0.766, recall=0.700, mAP50 = 0.727). Expert ratings averaged 4.25/5. Only 3.68% of images were rated as inappropriate due to false negative or false positive segmentations.

**Conclusion:**

ResNet18 accurately classified microscopic images for fungal elements, while the YOLOv11 model effectively delineated *Candida* morphologies, including yeasts, budding yeasts, and filamentous forms from clinical specimens. The high accuracy and positive expert feedback demonstrate the feasibility of integrating AI-assisted mobile microscopy into routine workflows, thereby advancing digital analysis of microbial infections using conventional light microscopy. With further clinical validation and expansion to include other infections, this approach holds great potential to establish robust real-world utility.

## 1 Introduction

Microscopic examination plays a central role in the clinical identification of microbial pathogens from patient-derived specimens. However, camera-equipped microscopes remain unavailable in many research facilities, large teaching laboratories, and healthcare centers, particularly in underserved areas, limiting access to digital image analysis and remote consultation. The widespread availability of mobile devices offers a practical, low-cost alternative for capturing high-resolution microscopy images. For example, mobile microscopy was successfully integrated into telemedicine for parasitic infection quantification in remote areas and into medical education through real-time image sharing and feedback (Dacal et al., 2021; Xu et al., 2025). At our institution, mobile microscopy is routinely used in teaching laboratories for reports, enhancing hands-on learning and mirroring real-world applications. This growing reliance on mobile devices presents an opportunity to overcome the limitations imposed by the lack of camera-equipped microscopes, enabling digital workflow integration and remote consultation within routine microscopic practice.

Vaginal discharge syndrome results from alterations in vaginal secretions, commonly due to infection of the vagina or cervix (Workowski et al., 2021) (Shroff, 2023). Most symptomatic cases of vaginal infections are caused by bacterial vaginosis (40–50%), vulvovaginal candidiasis (VVC; 20–25%), or trichomoniasis (15–20%) (Sobel, 1997; Paladine and Desai, 2018). Cervicitis, which frequently occurs secondary to sexually transmitted infections, may also present with vaginal discharge in conjunction with other clinical manifestations. Notably, *Neisseria gonorrhoeae* and *Chlamydia trachomatis* account for 30% to 50% of infectious cervicitis case (Shroff, 2023). Importantly, abnormal vaginal discharge must be distinguished from physiological vaginal discharge associated with normal vaginal conditions, which are characterized by a predominance of *Lactobacillus* species adherent to vaginal epithelial cells. Therefore, the ability to accurately distinguish among these various infections and non-infectious conditions is crucial for the effective clinical management of vaginal discharge syndrome (Shroff, 2023; Workowski et al., 2021).

Given the wide range of potential causes of vaginal discharge, this study focuses on VVC. Most women experience at least one episode, and up to 45% develop recurrent disease (Anderson et al., 2004; Sobel, 1997; Gonçalves et al., 2016). VVC typically presents with pruritus, soreness, vulvar erythema, and thick white discharge. Accurate *Candida* identification is essential to prevent misdiagnosis, persistent symptoms, and inappropriate treatment. Microscopic examination remains central to diagnosis, with Gram staining considered particularly reliable for distinguishing infection from commensal colonization (Qi et al., 2021). *Candida albicans* exhibits polymorphism, appearing as yeast, pseudohyphae, or true hyphae, with filamentous forms closely associated with virulence (Sudbery, 2011; Sudbery et al., 2004; Noble et al., 2017; Bettauer et al., 2022). Yeast cells alone may represent asymptomatic colonization, whereas budding yeasts or (pseudo)hyphae are required for laboratory confirmation of VVC (Workowski et al., 2021; Wang et al., 2025). Therefore, effective clinical diagnosis relies on the recognition of the various morphological forms of *Candida* species (Roselletti et al., 2019; Wang et al., 2025).

Recent advances in AI, particularly Convolutional Neural Networks (CNNs), have enabled automated fungal classification, detection, and segmentation (**Supplementary Table 1**) (Kim et al., 2024). However, most prior studies relied on high-quality images acquired under controlled conditions, using camera-equipped microscope, which limits their generalizability (**Supplementary Table 1**) (Kim et al., 2024). In real-world resource-limited settings, mobile-acquired images tend to display lower and more variable quality. Moreover, few studies have evaluated performance on images captured by non-experts such as medical students or frontline healthcare personnel. Robust models tailored to these conditions are therefore needed.

To address this gap, this study developed and validated deep learning models for fungal analysis using Gram-stained vaginal discharge samples acquired by mobile devices under realistic conditions. Our framework includes a ResNet18 classification model, a YOLOv5 detection model, and a YOLOv11 segmentation model. Our deep learning models can directly identify fungal morphology from a wide range of Gram-stained vaginal discharge smears, enabling timely and appropriate treatment decisions. By emphasizing heterogeneous imaging conditions and practical deployment, this work aims to bridge advanced AI techniques with routine mycology practice that may help reduce antifungal drug resistance and improve patient outcomes.

## 2 Materials and methods

### 2.1 Microscopic slide preparation of vaginal discharge specimens

Permanent Gram-stained slides of vaginal discharge were prepared for use in teaching laboratories. Vaginal discharge samples were collected anonymously from patients at Maharaj Nakorn Chiang Mai Hospital (Chiang Mai, Thailand) in accordance with relevant guidelines for human research, with approval from the institutional ethics committee (Approval No. 441/2568).

### 2.2 Study design and image collection

In total, 1,259 microscopic images of Gram-stained vaginal discharge were collected, encompassing the full spectrum of conditions seen in vaginal discharge syndrome. Specifically, the dataset included cases of bacterial vaginosis (BV), gonococcal infection (GU; *Neisseria gonorrhoeae*), chlamydial infection (NGU; *Chlamydia trachomatis*), trichomoniasis (TV; *Trichomonas vaginalis*), vulvovaginal candidiasis (VVC), as well as physiological (normal) vaginal samples dominated by *Lactobacillus*. This heterogeneous collection was used to train deep learning models for three tasks: binary classification of fungal presence (ResNet18), fungal object detection (YOLOv5), and fungal instance segmentation (YOLOv11). The number of images used for training each model is detailed in **Table 1**. An independent test set smartphone-captured images (not used in model training) was set aside to evaluate the final performance of each model (**Table 1**).

**Table 1:** Datasets used for the ResNet18, YOLOv5 and YOLOv11 models.

Dataset	ResNet18	YOLOv5	YOLOv11
Training set	570 (65%)	Set 1: 271 and Set 2: 244 (67%)	362 (60%)
Validation set	143 (17%)	Set 1: 32 and Set 2:62 (12%)	91 (15%)
Test set	159 (18%)	165 (21%)	165 (25%)
Total	872	774	618

Of the 1,259 mobile-acquired images, 894 were collected during a medical school laboratory session (Diagnostic of Vaginal Leucorrhea and Sexually Transmitted Infections, course 330329 PMLI II) from September to November 2025 (**Tables 2**, **3**). A total of 235 students each captured images from three different slides under 1000X magnification using their smartphones or tablets held to the microscope ocular (**Supplementary Figure 1A**). Students submitted images (.jpg or.heic format) via the university’s learning management system as routine lab reports. All images were downloaded, anonymized, and converted to .jpg format.

**Table 2:** Characteristics and number of images used in this study.

Type	Image #	Source
Mobile device Mobile device Camera-equipped microscope Total	894 298 67 **1259**	PMLI II Lab Research Lab Research Lab
Camera-equipped microscope Camera-equipped microscope Total	266 421 **687**	Roboflow (YOLOv5) Nguyen et al., 2025

**Table 3:** Number of images categorized by infections.

Group	BV	TV	GU	NGU	VVC	Transition	Healthy
F (n = 362)	–	–	–	–	28	188	146
NF (n = 351)	152	44	83	72	–	–	–

*There are 146 yeast-containing images (only yeast forms present) and 216 pseudohypha-containing images (at least one pseudohyphal form present, regardless of other morphologies). Of the pseudohypha-containing images, 28 lack Lactobacillus. Based on fungal morphology and Lactobacillus presence (**Supplementary Table 2**), the dataset includes 146 healthy images, 188 transition images, and 23 VVC images.

An additional 298 images were acquired in a controlled setting using a Celestron NexYZ universal adapter (Celestron, LLC; https://www.celestron.com) mounted on microscope eyepieces, allowing smartphones to function as digital microscopes (**Supplementary Figure 1B**). To replicate student image variability, at least two laboratory personnel captured images using three smartphone models (iPhone 8, 13, 15) on three Olympus CX31 microscopes. A subset of 67 images was also captured using a dedicated digital microscope (Nikon DS-Fi1 microscope model) to provide high-quality training and validation samples.

### 2.3 Image annotation

All images were annotated and reviewed by at least two medical mycology experts based on established diagnostic characteristics (**Supplementary Table 2**). Annotation involved drawing bounding boxes and free-form segmentation masks around fungal elements using the Labelbox platform (Labelbox, Inc.). A portion of images (10%) was annotated independently by at least two labelers to ensure consistency. One expert annotator (T.W.) then reviewed and reconciled any discrepancies to establish a consensus ground truth across all annotations. The finalized annotations were exported from Labelbox in JSON format and converted to the required YOLOv5 and YOLOv11 training formats using custom Python scripts. For the YOLOv11 segmentation format, pixel masks were converted into polygon coordinates using OpenCV’s approxPolyDP function with an epsilon value of 0.001, enabling the model to utilize polygon mask labels.

### 2.4 Image preprocessing

For the classification task, Contrast Limited Adaptive Histogram Equalization (CLAHE) was applied to improve image quality (Yadav et al., 2014; Lidong et al., 2015). CLAHE enhances local contrast and can reveal subtle features while minimizing noise amplification, thus facilitating more accurate detection and segmentation of fungal elements.

### 2.5 Model architecture and training

#### 2.5.1 Classification (presence/absence of fungus)

For binary classification, multiple CNN architectures were evaluated for efficient mobile deployment. MobileNetV2 (Sandler et al., 2018), EfficientNetB0 (Tan and Le, 2019), and ResNet18 (He et al., 2016) (all ImageNet-pretrained) were selected as candidates due to their compact size and fast inference. ImageNet is a widely used, large-scale visual database (https://www.image-net.org/) containing over 14 million annotated images across more than 20,000 categories. Each model was fine-tuned to classify images as fungus-positive or fungus-negative. ResNet18 achieved highest validation accuracy and was selected as the final classification model.

A ResNet18 CNN classifier was then trained to categorize images as containing fungal elements (F) or not (NF). The F group included samples with *Candida* (either VVC cases or asymptomatic *Candida* colonization; n = 362 images), while the NF group comprised samples from BV, TV, GU, and NGU, reflecting other causes of discharge (n = 351 images) (**Table 3**). The network used ImageNet-pretrained weights with the final layer modified for binary classification. Images were resized to 224×224 pixels with standard ImageNet normalization. Data augmentation during training involved resizing, random horizontal flipping (probability 0.5), and random rotation up to ±10° for each image per epoch. These transformations did not increase the number of images but enhanced dataset diversity throughout training. Training used Adam optimizer (learning rate 1×10^-4^), cross-entropy loss, and early stopping (patience=5 epochs). The best model was selected based on validation accuracy.

Model performance was evaluated on validation and independent test sets (159 images). The best checkpoint was loaded for inference. Test images underwent identical normalization and resizing as training. Predictions used softmax over two outputs, classifying images as F or NF based on higher probability. Standard metrics (precision, recall, F1-score, accuracy) were calculated using scikit-learn. Confusion matrices and ROC curves visualized performance. Gradient-weighted Class Activation Mapping (Grad-CAM++) (Selvaraju et al., 2017; Chattopadhay et al., 2018) was applied to representative images, generating heatmaps highlighting influential regions for predictions. All code was implemented in PyTorch and executed in Google Colab with GPU acceleration.

#### 2.5.2 Object detection and segmentation

For fungal object detection and segmentation, YOLO-based models were employed. Specifically, YOLOv5 (for detection) and YOLOv11 (for segmentation) models, pretrained by Ultralytics on the COCO (Common Objects in Context) dataset (Lin et al., 2014), were used as starting points. YOLOv5 was chosen for its lightweight, fast architecture, suitable for eventual mobile deployment, while YOLOv11 was used to showcase instance segmentation of fungal elements on mobile-acquired images.

For YOLOv5 detection, the small variant (YOLOv5s) was fine-tuned starting from the *bestYeast701.pt* model (Wangsanut et al., 2025), previously trained on general yeast and pseudohyphae images (https://universe.roboflow.com/yeast-rrffa/yeast-5arid/dataset/1). Although this base model recognized fungal morphology, it was not specialized for mobile-acquired Gram-stained images. The model was therefore fine-tuned on the 609-annotated mobile dataset (**Table 1**), producing *bestM2.2.pt*. Since YOLOv5 requires bounding boxes, filamentous forms (hyphae) were annotated with multiple small boxes along their length to minimize background inclusion during training.

For YOLOv11, a two-stage training strategy was employed to address the domain gap between natural and microscopy images. First, YOLOv11 was trained on an external public dataset of wet mount preparation of vaginal discharge images (Nguyen et al., 2025). A total of 421 images (**Table 2**) were annotated for fungal elements (yeast and hyphae), converted to segmentation masks, then polygon format, yielding an initial model (*bestVVC1.pt*). This model was then fine-tuned on the mobile-acquired dataset (453 images, **Table 1**), producing the final *bestF1.1.pt* model. This transfer learning approach enabled the model to learn general fungal morphology from high-quality images before adapting to smartphone image variability. All images were resized to 640×640 pixels. Training used 100 epochs, batch size 8, with PyTorch implementation of YOLOv5/YOLOv11 (built-in augmentation) on Google Colab GPUs.

For YOLOv5 hyperparameter tuning, Ultralytics’ automated evolution method was used (https://docs.ultralytics.com/yolov5/tutorials/hyperparameter_evolution). This genetic algorithm iteratively optimized hyperparameters (learning rate, batch size, augmentation settings) based on performance. Evolution was performed on an HPE Apollo 6500 system with AMD EPYC 7742 CPU and NVIDIA A100 GPU. Both YOLO models were evaluated on validation sets at multiple intersection over union (IoU) thresholds (0.25, 0.50, 0.75) following established methodologies (Koo et al., 2021; Ren et al., 2025).

#### 2.5.3 Data separation strategy

To prevent data leakage, training and test datasets were separated at the patient level by design, based on non-overlapping data source, slide identity, and acquisition date. Slides collected during different laboratory sessions originated from different patients. Images acquired on 16 September and 2 October 2025 were used for training, whereas images from 14 October 2025 were reserved exclusively for testing. Research laboratory slides, which yielded 50–100 images per slide, were obtained from a separate patient cohort and were distinct from those used in the teaching laboratory.

#### 2.5.4 Calculation of intersection-over-union and Dice coefficient

To quantitatively evaluate segmentation performance at the pixel level, IoU and Dice coefficient were calculated between the predicted segmentation masks and the corresponding ground-truth annotations. For each image, the binary mask generated by the YOLOv11 model was compared with the manually annotated reference mask. IoU was defined as the ratio of the overlapping area between the predicted and ground-truth masks to their union, while the Dice coefficient, which emphasizes spatial overlap, is commonly used for biomedical image segmentation. IoU and Dice scores were computed for each image and averaged across the test dataset. These metrics were applied consistently to both the internal mobile device–acquired images and the external open-source images to enable direct comparison of segmentation performance across datasets.

#### 2.5.5 Expert evaluation of the segmentation model

The YOLOv11 segmentation model was evaluated by an expert panel of clinicians and microbiologists specializing in medical mycology, each with >5 years of experience (**Supplementary Tables S3**-**S5**). Thirty-four images were selected: 19 positive (containing fungi) and 15 negative cases. YOLOv11-predicted segmentation masks were overlaid on original images and presented to experts. Each expert independently assessed: (1) appropriateness of predicted segmentation (whether highlighted areas matched actual fungal elements), and (2) satisfaction with model performance. Responses used a five-point Likert scale. A total of 272 ratings were collected (34 images × 8 experts). The frequency of “very inappropriate” ratings was recorded to identify failures. Rating distributions were analyzed separately for positive and negative images. Experts reported whether inaccuracies would impact clinical use (from “no impact” to “major deterrent”) and provided overall satisfaction ratings (1=very dissatisfied to 5=very satisfied).

## 3 Results

### 3.1 Classification task (presence vs absence of fungus)

**Figure 1:**
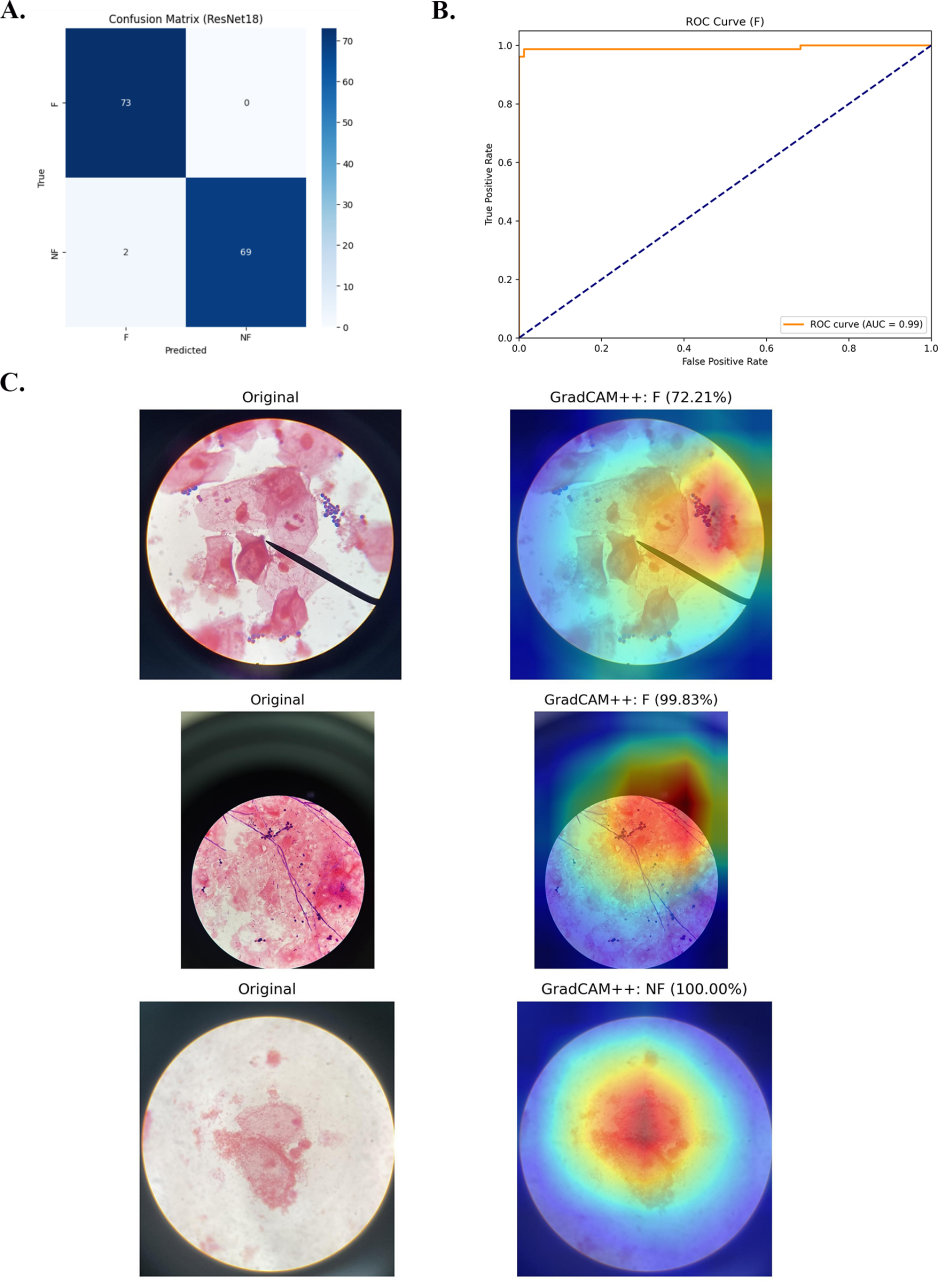
Confusion matrix, Receiver-Operating characteristic (ROC) curve, and Grad-CAM illustrate the classification performance of ResNet18 model on mobile-acquired microscopic images of Gram-stained vaginal discharge samples. **(A.)** The confusion matrix of model trained using pre-trained architecture: ResNet18. **(B.)** The ROC curves for the F (left) and NF (right) classes produced by the ResNet18 classification model on test images (n = 159). The area under the curve (AUC) is annotated for each class. **(C.)** Example of mobile device-microscopic images and the corresponding explanatory maps. Abbreviations: F, The presence of fungal elements, and NF, The absence of fungal elements.

To fully represent the spectrum of vaginal discharge syndromes encountered in clinical settings, the mobile device-acquired images included cases of VVC, BV, TV, GU, NGU, as well as physiologic discharge associated with a normal vaginal microbiome. All fungal morphologies, including yeast, budding yeast, and hyphae, were grouped into a single “fungus” category for classification and detection in this study; this approach was adopted for proof-of-concept purposes. On the independent test set of 159 unseen images, ResNet18 demonstrated superior performance among evaluated CNN architectures for binary classification, achieving an F1-score of 0.986 and AUC of 0.99 in distinguishing fungal from non-fungal images (**Figures 1A, B**; **Table 1**). Alternative models showed lower performance: MobileNetV2 and EfficientNetB0 achieved F1-scores of 0.882 and 0.889, respectively (**Supplementary Figure 2**; **Table 4**).

**Table 4:** Comparison of classification and object detection models for fungal recognition in Gram-stained vaginal discharge samples.

Model	Precision	Recall	F1 score
Classification			
MobileNetV2 - F - NF Average	0.8684 0.8971	0.9041 0.8592	0.8859 0.8777 0.8819
EfficientNetB0 - F - NF Average	0.8608 0.9231	0.9315 0.8451	0.8947 0.8824 0.8889
ResNet18 - F - NF Average	0.9733 1.0000	1.0000 0.9718	0.9865 0.9857 0.9857
Detection			
YOLOv5 Fungal elements	0.812	0.622	mAP50 = 0.730
Segmentation			
YOLOv11 Fungal elements	0.766	0.700	mAP50 = 0.727

F, The presence of fungal elements, NF, The absence of fungal elements, mAP50 = Mean average precision calculated at an intersection over union (IoU) threshold of 0.50.

**Figure 2:**
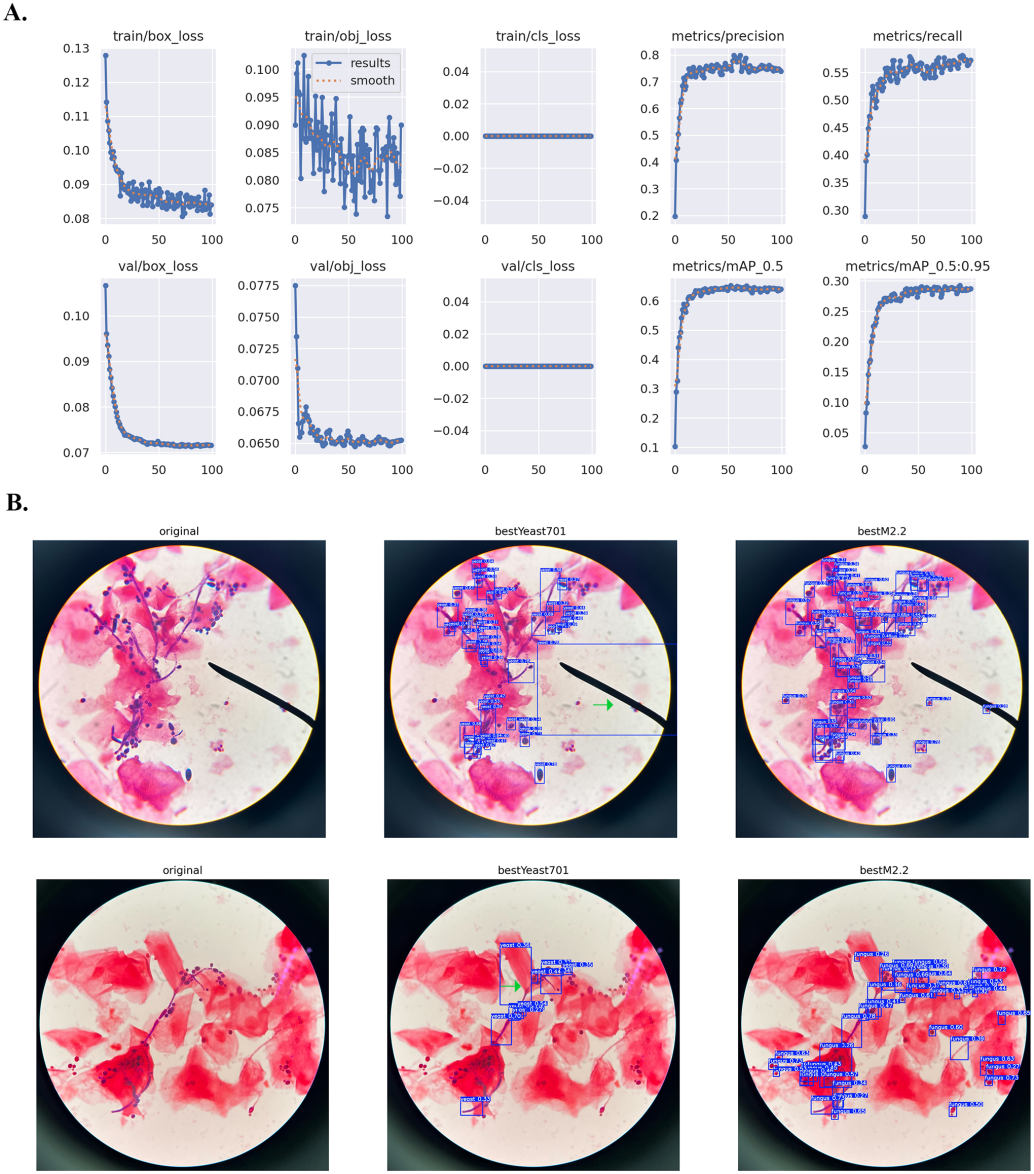
YOLOv5 training with transfer learning for mobile-acquired microscopic images of Gram-stained vaginal discharge samples. The bestYeast701.pt (yolov5s.pt-based model available via Hugging Face) was used as a base model and fine-tuned on mobile-acquired microscopic images using a transfer learning method, resulting in bestM2.2.pt model. **(A)** Training matrices from fine-tuning bestYeast701.pt to mobile-acquired images. **(B)** Visualization of model prediction after fine-tuning. Green arrow indicates background artifacts.

**Figure 3:**
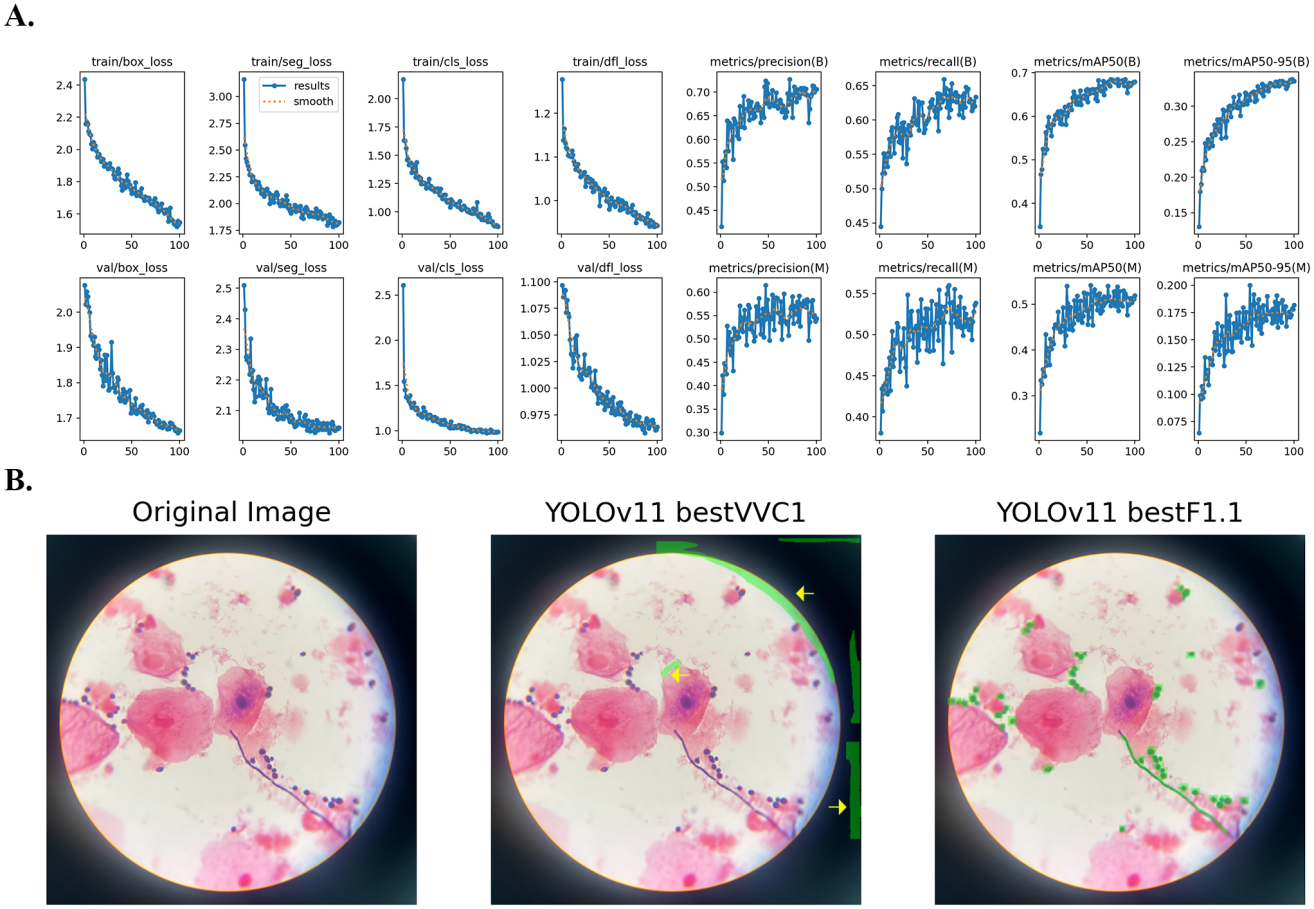
YOLOv11 training with transfer learning for mobile-acquired microscopic images of Gram-stained vaginal discharge samples. The yolov11s-seg.pt model was used as a base model and fine-tuned on mobile-acquired microscopic images using a transfer learning method, resulting in bestF1.1.pt model. **(A)** Training matrices from fine-tuning YOLOV11 segmentation model to mobile-acquired microscopic images with manual annotation of fungal elements (bestF1.pt base model). **(B)** Visualization of model prediction after fine-tuning, confident threshold was set to 0.1 for display fungal elements. Yellow arrow indicates background artifacts.

**Figure 4:**
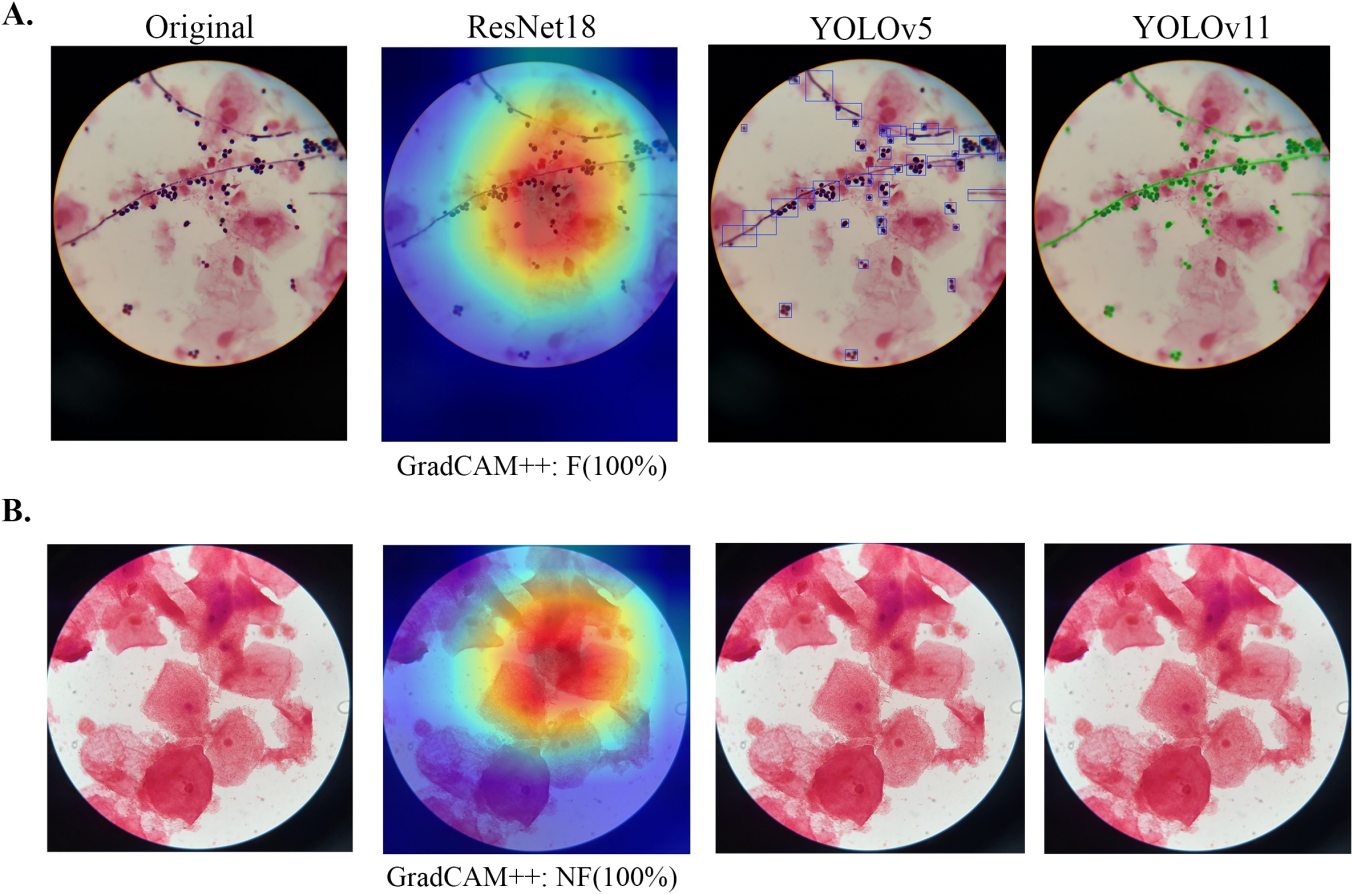
Visualization of fungal recognition on fine-tuned models using mobile device-acquired images of Gram-stained vaginal discharge samples. Images were acquired by using a smartphone **(A)** or a tablet **(B)**. The examples illustrate mobile device-acquired images that were correctly predicted as showing the presence (F, A.) or the absence (NF, B.) of fungal elements by ResNet18 classification (left panel), YOLOv5 fungal detection, (version M2.2 middle panel), and YOLOv11 fungal segmentation (version F1.1 right panel) in agreement with expert human ground truth.

Examples of prediction results on test images are illustrated in **Figures 1**-**4**. The YOLO models have been sequentially trained twice with human annotated images (first with conventional camera-acquired microscopic images and second with mobile device-acquired microscopic images), and then fine-tuned hyperparameter. The precision, recall and mAP50 values for the YOLOv5 and YOLOv11 models were reported at IoU of 0.50 and 0.25, respectively.

To generate visual explanation maps for ResNet18 models, Grad-CAM++ was applied to produce heatmaps highlighting the regions of input images that contributed most significantly to the model’s predictions. As depicted in **Figure 1C**, heatmaps consistently highlighted fungal cell clusters in *Candida*-positive images, demonstrating model focus on relevant microscopic features. In positive (F) images, overlays corresponded to yeast clusters or hyphae regions, while in negative (NF) images, attention was diffusely distributed or focused on background elements.

### 3.2 Fungal detection with YOLOv5

While the yeast forms of *Candida* species exhibit round to oval shapes, their filamentous forms appear as curved linear structures, which can pose challenges for fungal image recognition (Nguyen et al., 2025; Wang et al., 2025). As the YOLOv5 model detects objects using bounding boxes, filamentous form was manually annotated by drawing multiple small boxes along the length of each hypha to minimize background presented in vaginal discharge samples. A total of 609 microscopic images were manually annotated for the presence of fungal elements (**Tables 1**, **2**). To reduce the false-negative rate and ensure the model detects as many suspected hyphal regions as possible, we evaluated segmentation performance across a range of IoU thresholds (0.1, 0.25, 0.5, and 0.75), following recommendations from Koo et al. (2021) (**Table 3**). After fine-tuning, YOLOv5 demonstrated strong object-detection performance, achieving optimal results at IoU=0.50 with precision=0.812, recall=0.622, and mAP50 = 0.730 (**Tables 1**, **5**). At the more lenient IoU=0.25, precision remained high (>0.80) with increased recall, while at the stricter IoU=0.75, recall decreased despite maintained precision. IoU=0.50 was selected as the optimal threshold, balancing localization accuracy and false positive minimization.

**Table 5:** Performance of object detection and segmentation models across IoU thresholds.

IoU	Box Precision	Box Recall	Box mAP50	Mask Precision	Mask Recall	Mask mAP50
YOLOv5 0.10 0.25 0.50 0.75	0.758 0.743 0.812 0.750	0.687 0.691 0.622 0.583	0.714 0.719 0.730 0.668	- - - -	- - - -	- - - -
YOLOv11 0.10 0.25 0.50 0.75	0.809 0.803 0.788 0.752	0.739 0.740 0.739 0.711	0.765 0.771 0.764 0.741	0.775 0.766 0.767 0.741	0.697 0.700 0.690 0.656	0.724 0.727 0.723 0.704

For model detection visualization, fine-tuning on mobile device-acquired microscopic images substantially improved detection accuracy. In one test image, the retrained YOLOv5 detected 57 fungal elements versus 49 by the pre-trained model (**Figure 2B**, top). The fine-tuned model also reduced false positives, better distinguishing fungi from artifacts such as epithelial cell edges or microscope pointer arrows (green arrows, **Figure 2B**, bottom). These improvements demonstrate how transfer learning with domain-specific annotations enhanced *Candida* cellular morphology recognition.

### 3.3 Fungal segmentation with YOLOv11

To address the challenge of image recognition of filamentous forms, the YOLOv11 segmentation model was trained to enable precise annotation of the curved, linear structures characteristic of fungal hyphae. A total of 874 microscopic images were manually annotated for the presence of fungal elements using free-form segmentation (**Tables 1**, **2**). As shown in **Figure 3**, the YOLOv11 segmentation model accurately outlined individual yeast and hyphal structures even in challenging, variably stained fields. By contrast, the initial YOLOv11 model (before fine-tuning on our mobile device-acquired microscopic data) often misclassified background artifacts as fungal elements. For example, as shown in **Figure 3B**, a piece of background elements (yellow arrow, **Figure 3B** middle panel) was mistakenly highlighted as a fungus by the initial model but correctly ignored by the fine-tuned model (**Figure 3B** right panel). On a validation set of 91 images containing 1,132 annotated fungal instances, YOLOv11 achieved optimal performance at IoU=0.25, with precision=0.766, recall=0.700, and mAP50 = 0.727 (**Tables 1**, **5**). When evaluated as bounding boxes (ignoring mask boundaries), precision exceeded 0.80 at IoU=0.25, indicating accurate localization of fungal objects.

Both YOLO models (v5 and v11) exhibited high precision (0.7–0.8), indicating that detected fungal elements were usually correct. However, recall was more modest (0.6–0.7), likely due to imaging challenges in the heterogeneous mobile-captured dataset. Missed detections occurred predominantly in out-of-focus or low-contrast regions, particularly at image peripheries where microscope fields often blur with smartphone or tablet cameras, while centrally focused regions showed reliable detection.

Despite imaging variability, YOLOv5 and YOLOv11 effectively localized and delineated fungal elements in mobile device-acquired microscope images, performing robustly even on blurred, low-contrast, or artifact-laden images (**Figure 4A**). Importantly, both models correctly handled fungus-negative cases by producing no detections, even with complex backgrounds such as white blood cell-dominated slides (gonococcal/chlamydial infections) or *Gardnerella*-coated clue cells (BV), demonstrating crucial clinical specificity (**Figure 4B**).

#### 3.3.1 Expert evaluation of the AI segmentation model

Expert evaluation focused on the fungal segmentation models, as this image recognition task offers the greatest potential for explainable AI. A total of 34 mobile device-acquired microscopic images were included, consisting of 19 images containing fungal elements and 15 images without fungal elements. First, mask-based overlap metrics were calculated on this test set. The YOLOv11 model achieved an average IoU of 0.49 and an average Dice coefficient of 0.65, indicating moderate segmentation accuracy at the pixel level (**Table 6**, **Supplementary Figure 3**). Second, The same set of images was independently reviewed by eight experts, resulting in 272 total ratings. Across all ratings, the rate of “very inappropriate” responses was notably low, with only 10 ratings (3.68%) out of 272 assigned to this category, and these were confined to only two images (**Supplementary Table 3**; **Supplementary Figure 4**). For the 19 positive images (152 ratings), expert evaluations indicated high model performance, with 49.34% of responses rated as “mostly appropriate” and 25% as “very appropriate” (**Supplementary Table 3**; **Supplementary Figure 5**). For the 15 negative images (120 ratings), all expert responses (100.00%) rated the absence of a predicted mask as “very appropriate,” demonstrating that the model correctly recognized the background in every case (**Supplementary Table 4**). These findings highlight the high overall appropriateness and reliability of the AI model’s segmentation performance.

**Table 6:** Mask-based segmentation performance of the YOLOv11 model on the internal mobile device-acquired test set (n = 34; number of fungal-positive image = 19).

Metric	*Mean ± SD
IoU	0.49 ± 0.11
Dice	0.65 ± 0.10

* Values are reported as mean ± standard deviation.

Experts noted that the model performed well overall, effectively identifying fungal elements with only minor instances of under- or over- segmentation. These segmentation area issues were not considered significant enough to deter clinical or educational implementation: 37.5% stated minor errors would not affect willingness to use the model, 25% reported slight impact, and none indicated strong deterrence (**Supplementary Table 5**). Experts suggested that the target area or fungal elements should be centered within the image, as peripheral regions were often out of focus and frequently exhibited missing fungal segmentation masks. Overall satisfaction was strongly positive. The mean satisfaction was 4.25/5.

Together, the quantitative mask-based metrics and expert evaluations support the overall reliability and appropriateness of the YOLOv11 model’s segmentation performance on our mobile device–acquired images.

#### 3.3.2 External evaluation and generalizability

In the absence of external clinical datasets of mobile device–acquired microscopic images, we performed a preliminary, proof-of-concept external evaluation using 21 openly available Gram-stained microscopic images of vaginal discharge (**Supplementary Table 6**) to test the YOLOv11 model (bestF1.1.pt). The model delineated pseudohyphae and yeast morphologies in images obtained from outside our institution (**Supplementary Figure 6**; Beder et al., 2025; Nambiar et al. 2021), achieving an average IoU of 0.44 and Dice score of 0.60, indicating limited but measurable generalizability. Some false-positive and false-negative predictions were observed. Segmentation performance was slightly higher on internal mobile-acquired images (IoU = 0.49, Dice = 0.65), consistent with model optimization for mobile microscopy. Together, these results demonstrate the feasibility of morphology-based fungal detection on external images while highlighting the need for future validation using clinically sourced, mobile-acquired datasets.

## 4 Discussion

In this proof-of-concept study, we demonstrated that AI-based image recognition is a promising technology for the automatic identification of fungal elements using mobile device-acquired microscopic images of Gram-stained vaginal discharge. Our classification model, ResNet18, exhibited strong performance, as reflected by high F1 scores and AUC values, both matrices exceeding 0.90. The performance of our model is comparable to previous classification models trained on high quality images obtained from camera-equipped microscopes (**Supplementary Table 1**; (Wang et al., 2025; Nguyen et al., 2025). In a previous study, Nguyen et al. (2025) employed the MobileNetv2 model to screen wet-prep vaginal discharge samples for vaginitis diagnosis. Additionally, Wang et al., 2025 applied YOLOv5 for the automated diagnosis of VVC from Gram-stained vaginal discharge samples. Notably, none of these studies utilized a fungal segmentation model to delineate fungal cellular morphology from microscopic images. While our YOLOv11 segmentation model may occasionally over- or under-segment fungal elements, expert evaluation demonstrated these outputs remain clinically acceptable. This human-in-the-loop assessment provides practical validation that minor imperfections do not substantially impair diagnostic utility, supporting real-world applicability. Overall, our approach offers a range of image recognition tasks (ResNet18, YOLOv5, and YOLOv11) for the automatic identification of *Candida* morphology using mobile devices coupled with a conventional light microscope.

To our knowledge, this is the first study to develop and validate deep learning models for recognizing fungal elements in microscopic images acquired directly via mobile devices under everyday conditions. Although smartphone photography of microscopic fields has been increasingly explored for various biomedical applications (Soleimani et al., 2025; Hui et al., 2025; Soe et al., 2025, Soe et al., 2023), translating mobile microscopy to clinical practice remains challenging due to the substantial variability in how images are captured. In real clinical and educational settings, smartphone and tablet microscope images are often taken freehand (without a stabilizing adaptor), resulting in inconsistent focus, illumination, magnification, and field alignment. These issues are compounded by the wide range of device models, camera specs, and microscope optics that might be used across different settings. Prior studies have mitigated these factors by using phone-to-microscope adaptors to stabilize image capture (Dacal et al., 2021). In contrast, our study intentionally embraced the variability of true point-of-care usage: images were generated by many individuals (medical students) using unassisted handheld devices during routine lab sessions. This has direct implications for clinical implementation in low- and middle-income countries, remote clinics, and other point-of-care environments where standard digital microscopy and expert mycologists may not be available. Importantly, leveraging ubiquitous mobile devices means our approach can be adopted without the need for specialized hardware. Our work has the potential to strengthen global health diagnostics by improving accessibility and bridging gaps in infrastructure and expertise.

It should be emphasized that detection of fungal elements by our current model does not equate to a clinical diagnosis; it does not differentiate between colonization and infection, nor does it quantify fungal burden in a clinically actionable way. Instead, these findings should be regarded as a screening aid to inform, but not determine, clinical decision-making. Clinicians are advised to interpret the results in conjunction with clinical context and further diagnostic workup. Future studies could expand this fungal detection foundation into broader diagnostic applications. We are currently developing a separate deep learning model designed to distinguish between healthy and infected states, as well as other causes of vaginal discharge syndrome. For instance, integrating our model with algorithms recognizing other vaginal discharge features—such as clue cells (BV), motile *Trichomonas* (trichomoniasis), or intracellular diplococci (gonorrhea)—could enable comprehensive vaginal infection screening. Additionally, combining fungal detection with *Lactobacillus* quantification could potentially improve diagnostic accuracy, as *Lactobacilli* are typically abundant in healthy samples but diminished in VVC. This multimodal approach could enhance discrimination between VVC, normal physiology, and other infections.

Beyond vaginal candidiasis, the trained model weights offer transfer learning potential for other fungal imaging tasks. The progressive training strategy—from high-quality to mobile images—produced robust features adaptable to different clinical contexts, such as detecting *Fusarium* or *Aspergillus* in corneal smears (fungal keratitis), or dermatophytes in skin/nail scrapings (ringworm, onychomycosis). Future research could leverage these models as starting points for other mycoses, potentially accelerating AI development across medical mycology applications.

We acknowledge several limitations in our study. First, the sample collection was from a single center and primarily involved images taken by students in an educational setting. While this provided a convenient diversity of image conditions, it may not capture the full variability of clinical practice (e.g., images from other hospitals or regions). Nevertheless, within a teaching context, our approach met its goal of exposing students to authentic clinical specimens and preparing them for real-world scenarios. Second, we only used Gram-stained slides; in practice, wet-mount (saline or KOH prep) microscopy is also common for diagnosing vaginal infections. Future work should include other preparation methods to broaden the model’s applicability. Third, our binary classification and detection tasks treated all fungal morphologies (yeast, budding yeast, hyphae) as one “fungus” category versus background. While this simplification was useful for an initial proof-of-concept, it does not differentiate between the forms of *Candida*. In future iterations, the framework could be refined to classify specific fungal morphologies or even identify species of *Candida* (Cuevas-Tello et al., 2025), given sufficient data. Transitioning this platform from an educational prototype to a clinical diagnostic tool will also involve collecting more image data that reflect patient-level variability (different patient populations, varying specimen types, use of both Gram stain and wet prep, etc.). Expanding the training data in this manner would likely improve the robustness, generalizability, and clinical utility of the deep learning models.

## 5 Conclusion

We present a pioneering deep learning framework for morphology-based recognition of fungal elements in mobile device-acquired microscopic images. The fungal classification, detection, and segmentation models demonstrate strong performance on images captured by students in medical training settings, indicating potential for clinical application. This work establishes a foundation for broader multimodal AI platforms to aid diagnosis of vaginal discharge syndromes and other fungal infections. Conventional light microscopes can be effectively integrated with smartphone or tablet cameras and AI algorithms, bridging traditional microscopy with modern digital diagnostics. This advancement is especially valuable in resource-limited environments, enabling applications that span from medical education to clinical diagnostics. Ultimately, our approach contributes to a practical solution that supports improved surveillance, patient care, and research of pathogenic fungi. Our work aligns closely with the collective goal of developing innovative tools and strategies to confront the growing global challenge of fungal infections.

## Data Availability

The datasets presented in this study can be found in online repositories. The names of the repository/repositories and accession number(s) can be found in the article/**Supplementary Material**.
