# Supplementary material for: Fungal recognition in vaginal discharge using deep learning analysis of mobile device-acquired microscopic images
Source: Front Cell Infect Microbiol. 2026 Mar 12;16:1787545. doi: 10.3389/fcimb.2026.1787545 (PMC13017809; doi:10.3389/fcimb.2026.1787545)
Supplement: Supplementary file 1 [file Image1.pdf]

A.

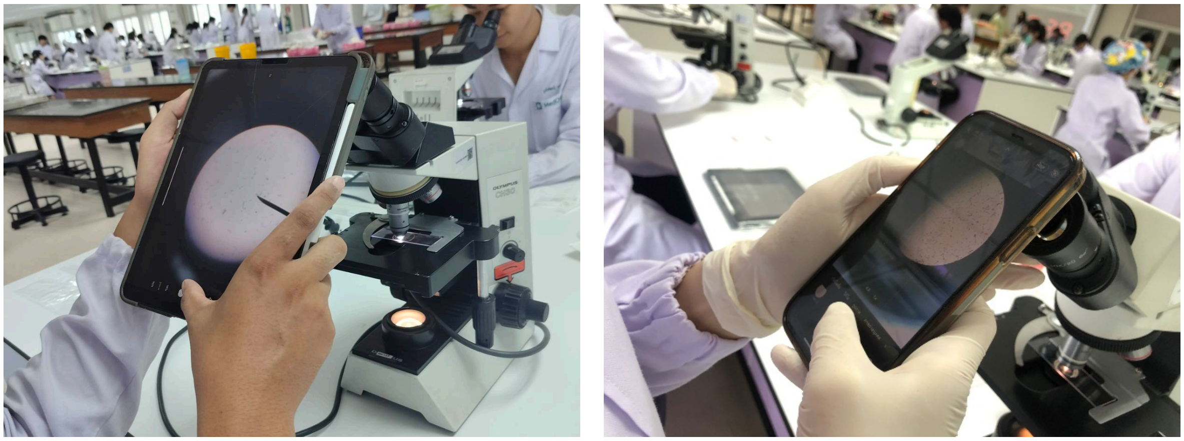

B.

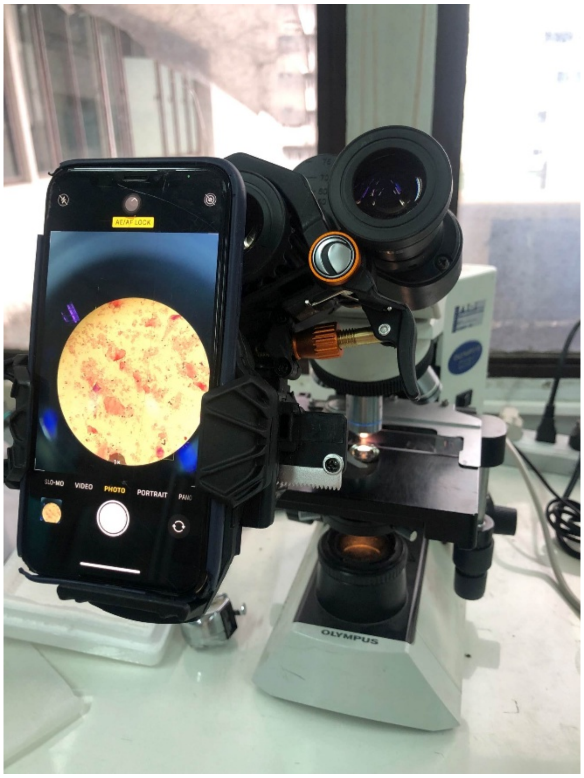

Figure S1. Microscopic images were acquired by mobile devices. A. Microscopic images were acquired by students during laboratory sessions (left: tablet, right: smartphone). **B**. Microscopic images were acquired using smartphone adaptor attached to the eyepiece of microscope.

A.

B.

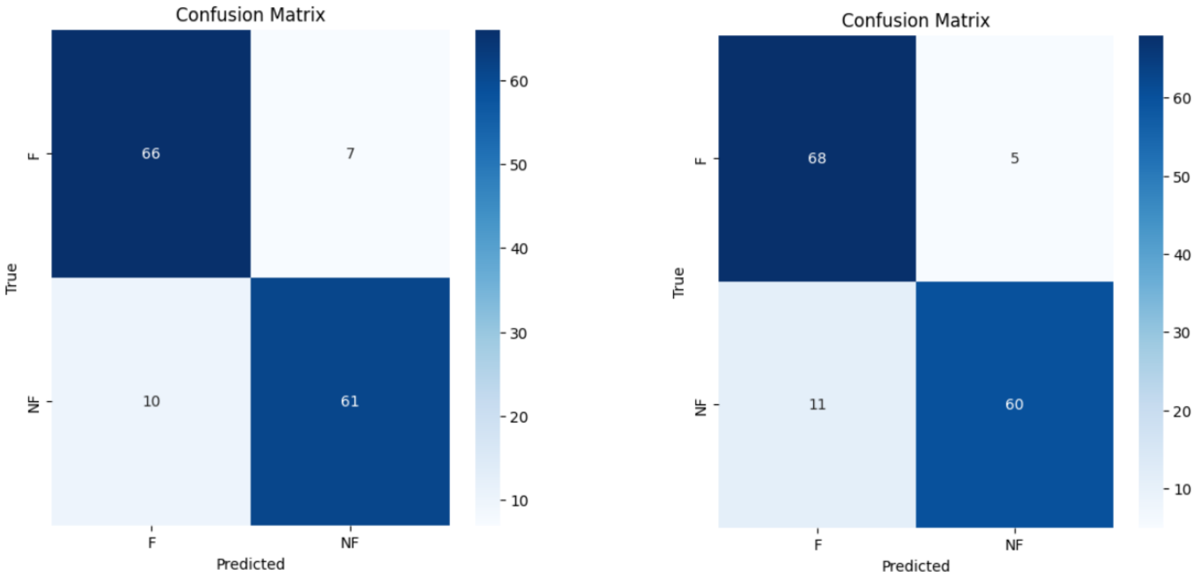

Figure S2. Confusion matrices illustrate the classification performance of various models on mobile-acquired microscopic images of Gram-stained vaginal discharge samples. The models were trained using pre-trained architectures: MobileNetV2 (**A**.) and EfficientNetB0 (**B**.). Abbreviations: F = The presence of fungal elements, and NF = The absence of fungal elements. Accuracy scores are presented in Table 3. Model training was performed using transfer learning on EfficientNetB0 and MobileNetV2 architectures, both pre-trained on ImageNet and implemented in TensorFlow. All images were resized to 224 × 224 pixels. The models were trained for 30 epochs with a batch size of 16 using an augmented dataset. Data augmentation included random rotations (up to 25°), width/height shifts (up to 20%), shear (up to 20%), zoom (up to 20%), and horizontal flipping, with empty areas filled by nearest pixel interpolation. Class weighting addressed data imbalance. Model performance was monitored on a validation set, and training was optimized with Keras callbacks such as early stopping to select the best-performing model.

A.

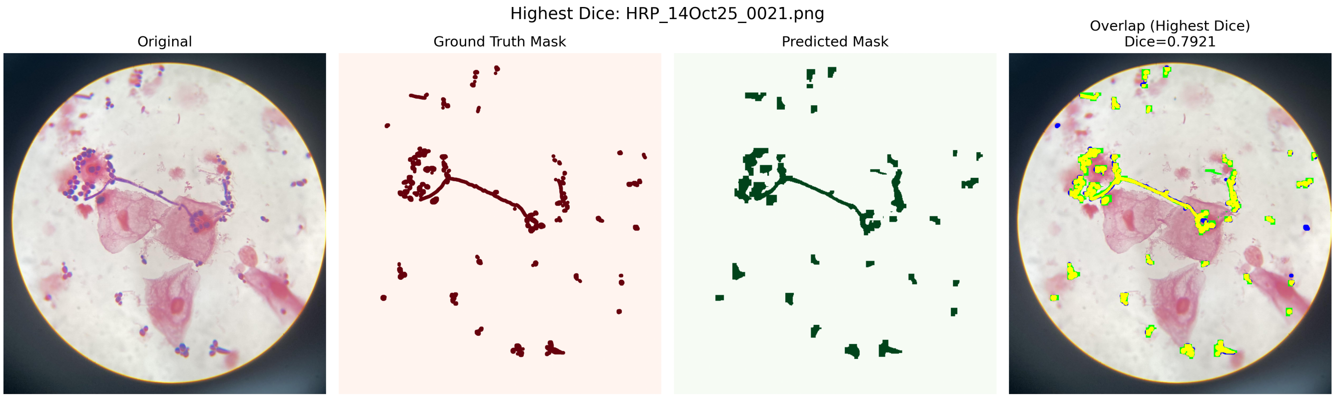

B.

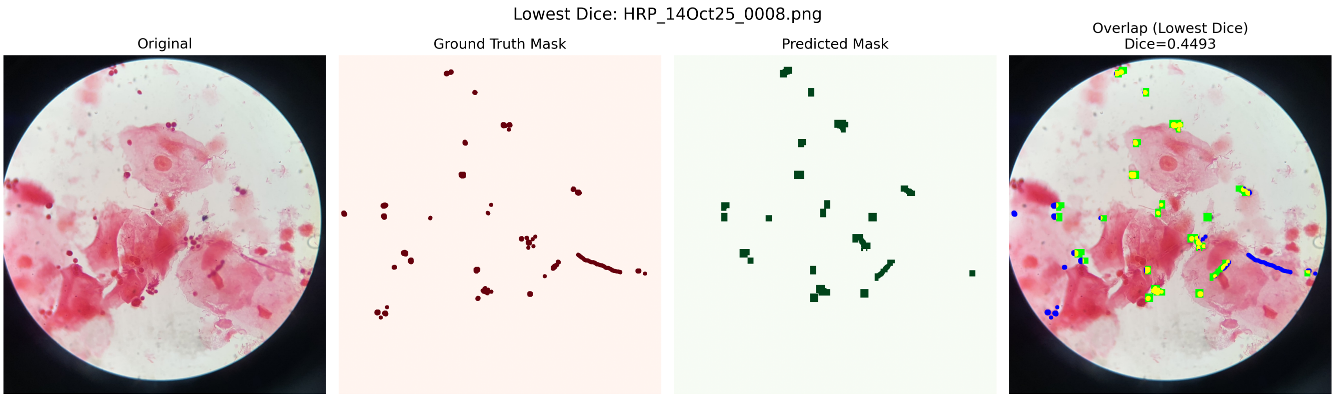

Figure S3. Ground-truth and predicted masks for the mobile device-acquired microscopic image test set. Dice scores were computed as described in the Materials and Methods. (A) Highest Dice score. (B) Lowest Dice score. n = 34 (fungal-positive = 19).

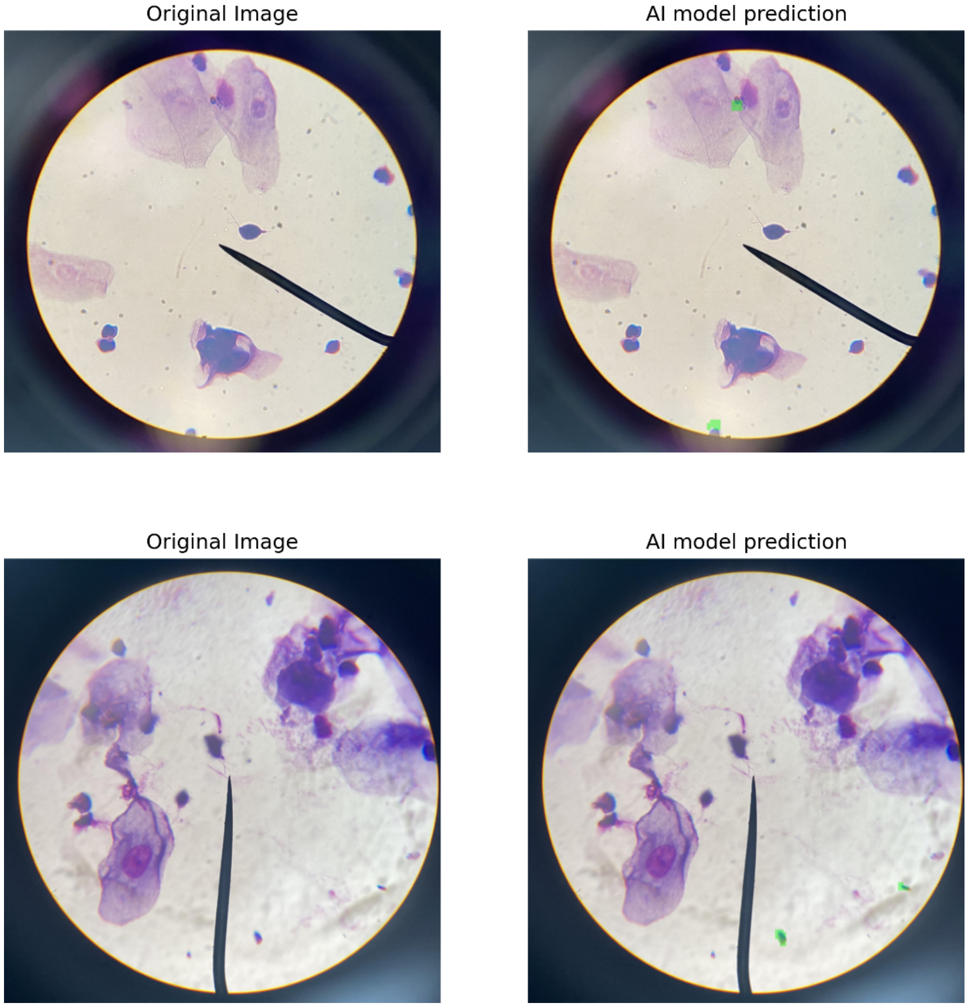

Figure S4. Two images rated as "very inappropriate to mostly inappropriate" by experts due to false positive or false negative results in the AI-generated fungal segmentation mask. In both cases, the ground truth diagnosis is *Trichomonas vaginalis* (TV).

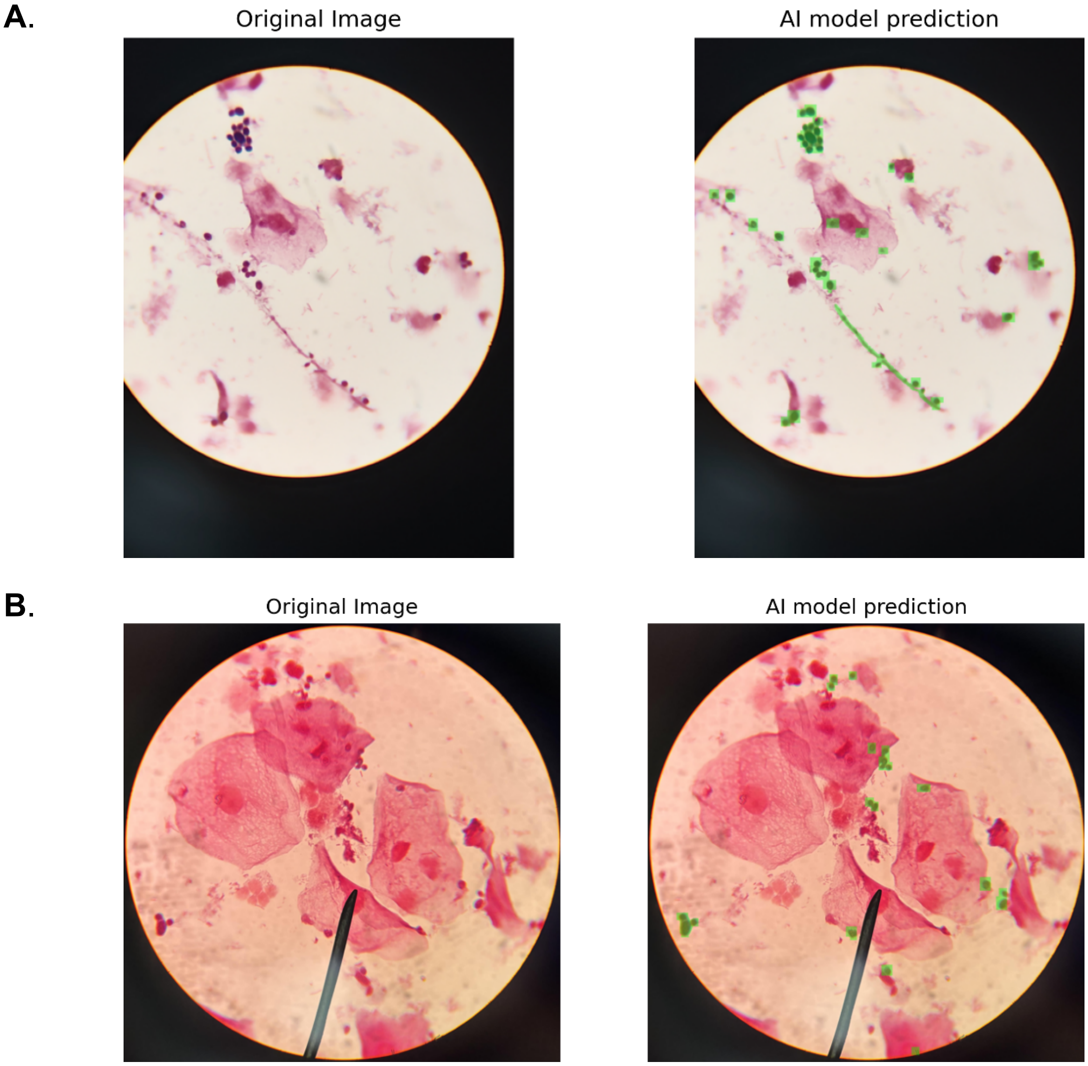

Figure S5. Representation of AI model prediction from expert evaluation eurvey. **A**. The AI model's prediction of fungal segmentation masks rated as "very appropriate" by 75% of experts (6 out of 8). **B**. The AI model's prediction of fungal segmentation masks rated as "moderately to mostly appropriate" by 87.5% of experts (7 out of 8).
