# Supplementary material for: Fungal recognition in vaginal discharge using deep learning analysis of mobile device-acquired microscopic images
Source: Front Cell Infect Microbiol. 2026 Mar 12;16:1787545. doi: 10.3389/fcimb.2026.1787545 (PMC13017809; doi:10.3389/fcimb.2026.1787545)
Supplement: Supplementary file 2 [file Image2.pdf]

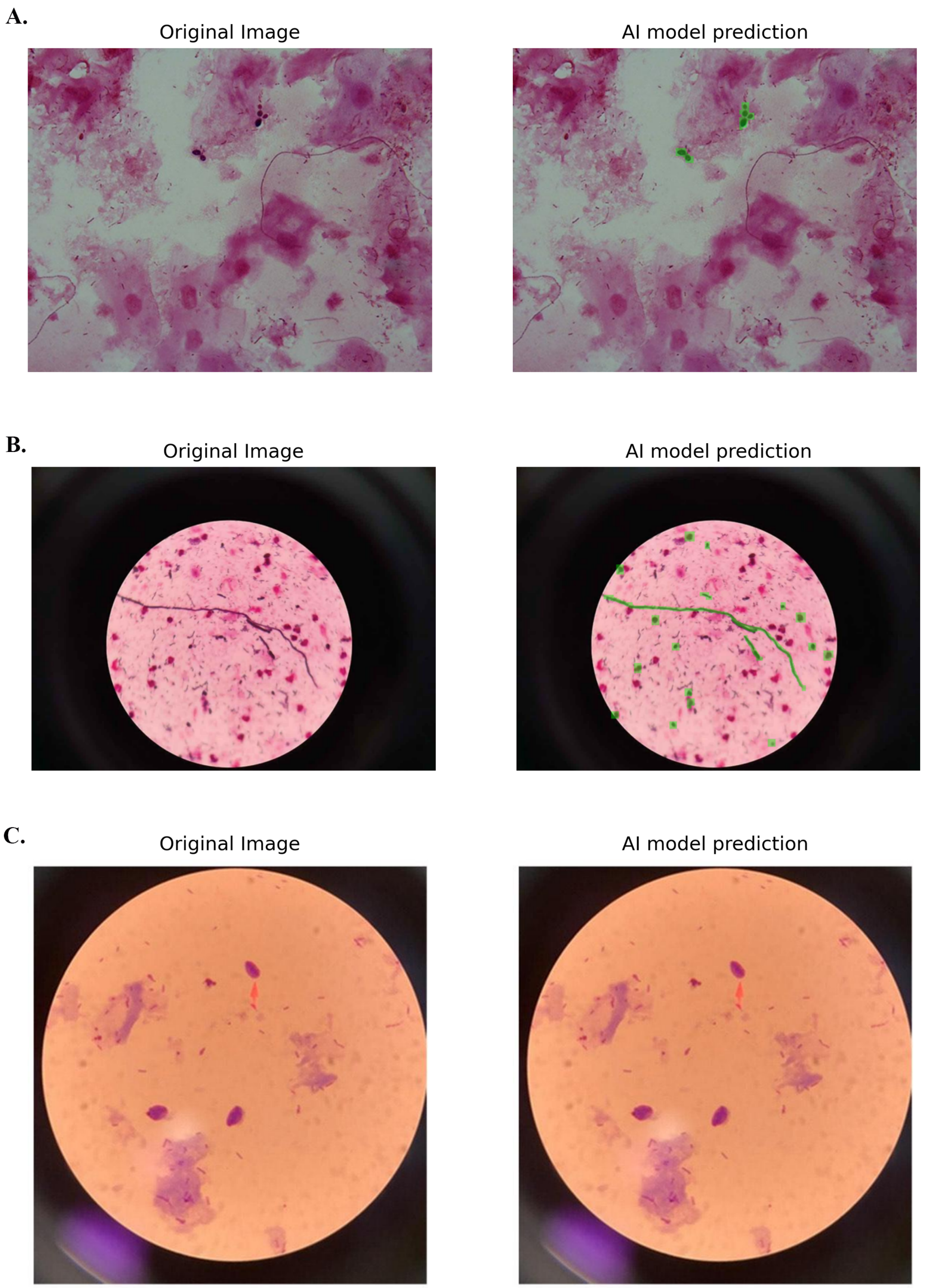
A.
Original Image
AI model prediction
B.
Original Image
AI model prediction
C.
Original Image
AI model prediction

Figure S6. External evaluation of the YOLOv11 fungal segmentation model using open-sourced microscopic images. Representative images from external sources (licensed under Creative Commons Attribution 4.0 [CC BY 4.0] or Attribution-ShareAlike 4.0 [CC BY-SA 4.0]) were used to evaluate the model's ability to delineate pseudohyphae and yeast morphologies. Model predictions are shown as green segmentation overlays on fungal positive image. (**A.**) Microscopic images of yeast cells, available at https://www.utas.edu.au/health/resources/open-resources/resources/courses/laboratory-medicine/cxa-342-medical-microbiology (CC BY-SA 4.0). (**B.**) Gram-stained microscopic image of oral candidiasis, showing budding yeast cells and pseudohyphae along with a few epithelial cells adapted from (Nambiar et al., 2021) (CC BY 4.0). (**C.**) Giemsa-stained microscopic image from *Trichomonas vaginalis*–positive patient, adapted from (Beder et al., 2025) (CC BY 4.0).
