# Supplementary material for: Fungal recognition in vaginal discharge using deep learning analysis of mobile device-acquired microscopic images
Source: Front Cell Infect Microbiol. 2026 Mar 12;16:1787545. doi: 10.3389/fcimb.2026.1787545 (PMC13017809; doi:10.3389/fcimb.2026.1787545)
Supplement: Supplementary file 3 [file Table1.docx]

**Table S1. Comparison of our fungal analysis models with related studies.**

| **Fungal type** | **Scope** | **Model(s)** | **Dataset** | **Ref** |
| --- | --- | --- | --- | --- |
| **Fungal infections** | | | | |
| Cutaneous fungal infection | Identification of spore and hyphae from superficial fungal infection. | Dual-model framework: YOLOX for spore detection and MobileNetV2 for mycelium classification | Fluorescence microscopic image | (Ren et al., 2025) |
|  | Classification of superficial fungal infection. | VGG16, Inception V3 and ResNet50 | DeFungi: Bright field microscopic image | (Sopo et al., 2021) |
|  | Classification of superficial fungal infection. | MeFunX | DeFungi: Bright field microscopic image | (Rawat et al., 2024) |
|  | Object detection of skin and nail fungal infection. | YOLOV4 | KOH Bright field microscopic image | (Koo et al., 2021) |
|  | Hyphal segmentation of dermatophytes in clinical samples | ResNet and attention-based modifications of U-Net | KOH Bright field microscopic image | (Rajitha et al., 2025) |
|  | Onychomycosis detection | VGG16 and Inception V3 | KOH Bright field microscopic image | (Yilmaz et al., 2022) |
| Vaginal fungal infection | Classification of vaginal infection | MobileNetV2 | KOH Bright field microscopic image | (Nguyen et al., 2025) |
|  | Object detection of vaginal candidiasis | YOLOV5 | Gram’s stain Bright field microscopic image | (Wang et al., 2025) |
| **Culture** | | | | |
| Contaminant fungi | Classification of 89 fungal genera that are common fungal pathogens | DenseNet, Inception ResNet,  InceptionV3, Xception,  ResNet50, VGG16, and VGG19 | Bright field microscopic image | (Rahman et al., 2023) |
|  | Classification of 6 mold species | Standard CNN | Bright field microscopic image | (Cighir et al., 2025) |
|  | Object detection of *Aspergillus* species | YOLOV8 | Bright field microscopic & Macroscopic image | (Hassan et al., 2025) |
|  | *Aspergillus* species identification | DenseNet-121, InceptionV3, and ResNet-18 | Macroscopic image acquired by digital camera and iPhone | (Tsang et al., 2025) |
|  | Segmentation of *Fusarium* spores | CRF_ResUNet++ | Image acquired by Electron microscope | (Zhang et al., 2024) |
| **Our work** | - Fungal classification, detection and segmentation of vaginal candidiasis | - MobileNetV2, EfficientNetB0, ResNet18 - YOLOv5, and YOLOv11 | Mobile device-acquired microscopic images | This work. |

**Table S2. Key characteristics observed in Gram-stained microscopic images of vaginal discharge samples for each class of vaginal infections.**

| **Infections** | **Common pathogens** | **Key characteristics** |
| --- | --- | --- |
| Bacterial Vaginosis | *Gardnerella* spp | Presence of clue cells. |
| Gonorrhea Urethritis | *Neisseria gonorrhoeae* | Many white blood cells with presence of intracellular Gram-negative diplococcic. |
| Non-Gonorrhea Urethritis | *Chlamydia* *trachomatis* | Many white blood cells but not detected visible bacteria. |
| Trichomonas Vaginosis | *Trichomonas* spp | Presence of trophozoites with flagella. |
| Vulvovaginal Candidiasis | *Candida* spp | Presence of budding yeast  cells and/or pseudohyphae. |

**Table S3.** Distribution of expert ratings for AI-predicted segmentation masks in images containing fungal elements (total images = 19 an total experts = 8). Each image containing green fungal segmentation masks was rated by 8 experts (total of 152 ratings).

| **Rating** | **Response (n)** | **% of Total** |
| --- | --- | --- |
| Very inappropriate (false positive and false negative) | 10 | 6.58 |
| Mostly inappropriate (mask covers much too large or much too small an area) | 10 | 6.58 |
| Moderately appropriate (mask covers more or less than ideal, but somewhat close) | 19 | 12.50 |
| Mostly appropriate (mask is close to ideal) | 75 | 49.34 |
| Very appropriate (mask closely matches the ideal area) | 38 | 25.00 |
| Total | 8 experts x 19 images = 152 | 100 |

**Table S4.** Distribution of expert ratings for AI-predicted segmentation masks in images without fungal elements (total images = 15, total experts = 8). Each image without green fungal segmentation masks was rated by 8 experts (total of 120 ratings).

| **Rating** | **Response (n)** | **% of Total** |
| --- | --- | --- |
| Very inappropriate (false negative) | 0 | 0.00 |
| Very appropriate (no mask because there are no fungal elements) | 120 | 100.00 |
| Total | 8 experts x 15 images = 120 | 100.00 |

| **Response** | **n** | **%** |
| --- | --- | --- |
| Do not affect my decision | 3 | 37.50 |
| Slightly affect my decision | 4 | 25.00 |
| Somewhat affect my decision | 1 | 37.50 |
| Strongly affect my decision | 0 | 0.00 |

**Table S5.** Distribution of expert responses to the potential impact of predicted segmentation areas for fungal elements (e.g., missed regions, significant over- or under-segmentation) on decision to implement this AI model in clinical or educational settings.

**Reference**

CIGHIR, A., BOLBOACĂ, R. & LENARD, T. 2025. OpenFungi: A machine learning dataset for fungal image recognition tasks. *Life (Basel),* 15.

HASSAN, H. M., AMIR, A., ABD EL-GHANY, M. N. M., SALIH, S. A. & OUF, S. A. 2025. *Aspergillus* detection based on deep learning model using YOLOv8 with a small custom dataset. *Egyptian Journal of Botany,* 65**,** 211-226.

KOO, T., KIM, M. H. & JUE, M. S. 2021. Automated detection of superficial fungal infections from microscopic images through a regional convolutional neural network. *PLoS One,* 16**,** e0256290.

NGUYEN, T. B., NGUYEN, H. B., LE, T. X., BUI, T. H., NGUYEN, L. S., NGUYEN, T. H. & NGUYEN, T. C. 2025. Applying machine learning with MobileNetV2 model for rapid screening of vaginal discharge samples in vaginitis diagnosis. *Sci Rep,* 15**,** 19171.

RAHMAN, M. A., CLINCH, M., REYNOLDS, J., DANGOTT, B., MEZA VILLEGAS, D. M., NASSAR, A., HATA, D. J. & AKKUS, Z. 2023. Classification of fungal genera from microscopic images using artificial intelligence. *J Pathol Inform,* 14**,** 100314.

RAJITHA, K., KRISHNAMOORTHY, A., PRAKASH, P., GOVINDAN, S., RAO, R. & PRASAD, K. 2025. Segmentation of microscopic images of dermatophytes in clinical samples using ResNet and attention-based modifications of U-Net. *Neural Computing and Applications***,** 1-17.

RAWAT, S., BISHT, B., BISHT, V., RAWAT, N. & RAWAT, A. 2024. MeFunX: A novel meta-learning-based deep learning architecture to detect fungal infection directly from microscopic images. *Franklin Open,* 6**,** 100069.

REN, R., TAN, W., CHEN, S., XU, X., ZHANG, D., CHEN, P. & ZHU, M. 2025. Deep learning application to hyphae and spores identification in fungal fluorescence images. *Sci Rep,* 15**,** 27222.

SOPO, C. J. P., HAJATI, F. & GHEISARI, S. 2021. DeFungi: Direct mycological examination of microscopic fungi images. *arXiv preprint arXiv:2109.07322*.

TSANG, C. C., ZHAO, C., LIU, Y., LIN, K. P. K., TANG, J. Y. M., CHENG, K. O., CHOW, F. W. N., YAO, W., CHAN, K. F., POON, S. N. L., WONG, K. Y. C., ZHOU, L., MAK, O. T. N., LEE, J. C. Y., ZHAO, S., NGAN, A. H. Y., WU, A. K. L., FUNG, K. S. C., QUE, T. L., TENG, J. L. L., SCHNIEDERS, D., YIU, S. M., LAU, S. K. P. & WOO, P. C. Y. 2025. Automatic identification of clinically important *Aspergillus* species by artificial intelligence-based image recognition: proof-of-concept study. *Emerg Microbes Infect,* 14**,** 2434573.

WANG, Z., WANG, R., GUO, H., ZHAO, Q., REN, H., NIU, J., WANG, Y., WU, W., LIANG, B., YI, X., ZHANG, X., XU, S., DONG, X., WANG, L. & LIAO, Q. 2025. AI-assisted diagnosis of vulvovaginal candidiasis using cascaded neural networks. *Microbiol Spectr,* 13**,** e0169124.

YILMAZ, A., GÖKTAY, F., VAROL, R., GENCOGLAN, G. & UVET, H. 2022. Deep convolutional neural networks for onychomycosis detection using microscopic images with KOH examination. *Mycoses,* 65**,** 1119-1126.

ZHANG, D., ZHANG, W., CHENG, T., LEI, Y., QIAO, H., GUO, W., YANG, X. & GU, C. 2024. Segmentation of wheat scab fungus spores based on CRF_ResUNet++. *Computers and electronics in agriculture,* 216**,** 108547.
