# Supplementary material for: Fungal recognition in vaginal discharge using deep learning analysis of mobile device-acquired microscopic images
Source: Front Cell Infect Microbiol. 2026 Mar 12;16:1787545. doi: 10.3389/fcimb.2026.1787545 (PMC13017809; doi:10.3389/fcimb.2026.1787545)
Supplement: Supplementary file 4 [file Table2.pdf]

Supplemental Table S6. External evaluation of model predictions (YOLOv11, bestF1.1.pt) using open-source microscopic images of Gram-stained vaginal discharge (n = 21). Model predictions (green segmentation mask) are overlaid on fungal positive-images. (+) indicates fungal-positive image and (-) indicates fungal-negative images. IoU and Dice scores are reported.

[a]Model prediction and reference	Ground truth	IoU	Dice
(Ramsay et al., 2009)	*C. albicans* (+)	0.61	0.76
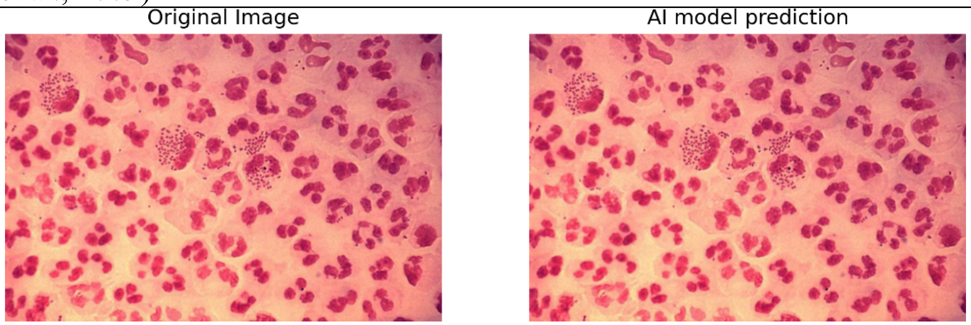<br>https://commons.wikimedia.org/wiki/File:Gonococcal_urethritis_PHIL_4085_lores.jpg	GU (-)		
http://thunderhouse4-yuri.blogspot.com/2010/11/bacterial-vaginosis.html	Yeast infection (+)	0.58	0.73
http://thunderhouse4-yuri.blogspot.com/2010/11/bacterial-vaginosis.html	*Mobiluncus* species (-)		
https://www.sciencephoto.com/media/295164/view/bacterial-vaginosis-cervical-smear	Bacterial vaginosis, cervical smear (-)		
https://www.scirp.org/journal/paperinformation?paperid=98692	Figure 4: non-bacterial type (-)		
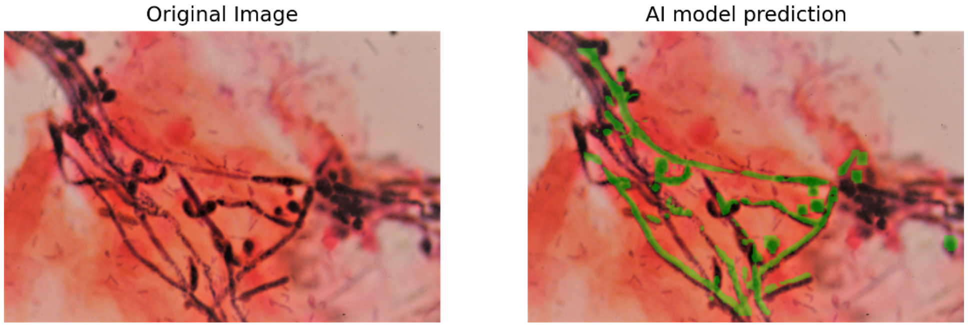<br>https://commons.wikimedia.org/wiki/File:Vaginal_candidiasis,_Gram_stain.jpg	Vaginal candidiasis (+)	0.31	0.48

https://www.facebook.com/photo/?fbid=1438298840031919&set=pcb.3280065225592310	BV (-)		
https://theobjective.com/sociedad/2025-09-04/candidiasis-vaginal-problema-medico-frecuente/	Vaginal candidiasis (+)	0.45	0.62
https://www.reddit.com/r/Sourdough/comments/1odgy1m/i_cultured_my_starter _update_gram_stain_photos/?captcha=1	Image #4: Yeasts (+)	0.37	0.54
https://gram-stain.com/wp-content/uploads/2016/06/20160411_110554269_iOS.jpg	*C. albicans* (+)	0.77	0.87
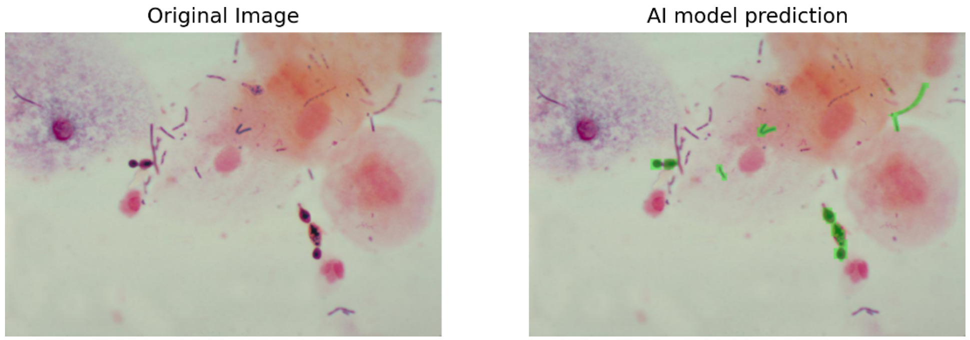<br><br>http://www.publicdomainfiles.com/show_file.php?id=13539954623936	*C. albicans* (+)	0.43	0.60
(McGarry, 2010)	Fig. 8.3. P. 81 Budding yeast and hyphae (+)	0.26	0.41
https://www.instagram.com/p/DCkc15aBca5/	Vaginal candidiasis (+)	0.21	0.34
https://microbiologylearning.weebly.com/candida-yeast.html	Budding Candida Yeast Cells and Pseudohyphae (+)	0.38	0.55
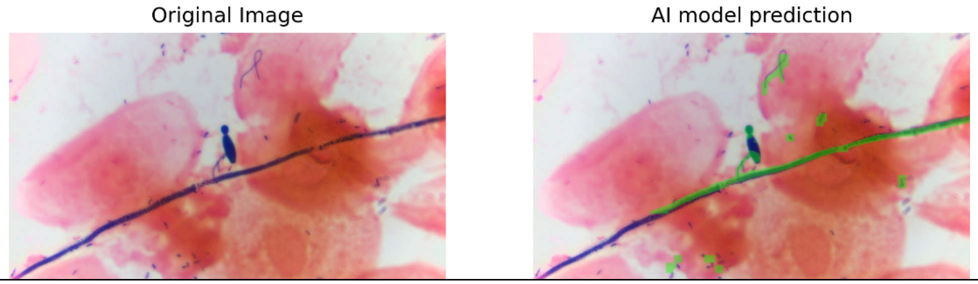 	*C. albicans* (+)	0.36	0.53

https://commons.wikimedia.org/wiki/File:Candida_albicans_-_Gram_stain.jpg			
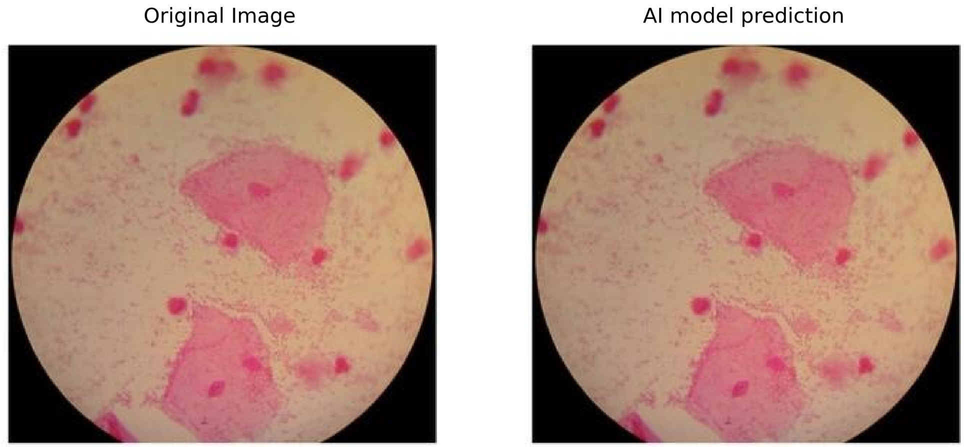<br><br>Adapted from<br>(Chudzicka-Strugała et al., 2024)	BV (-)		
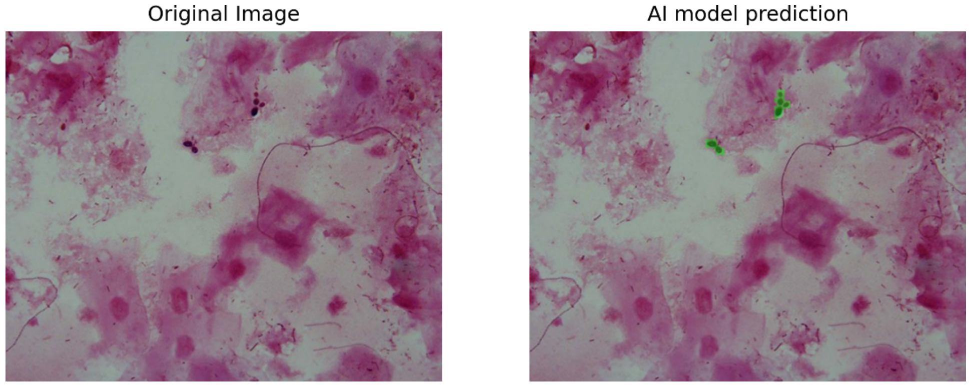<br><br>Figure 5A. in main manuscript<br><br>https://www.utas.edu.au/health/resources/open-resources/resources/courses/laboratory-medicine/cxa-342-medical-microbiology	Module answer 2:<br>*C. albicans* (+)	0.60	0.75

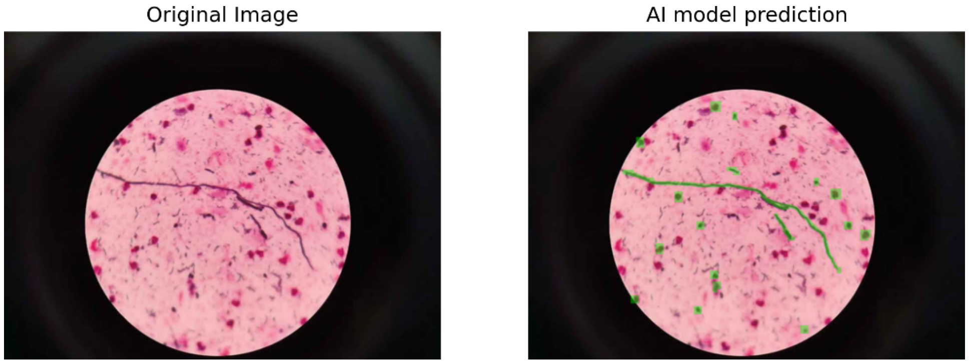<br>Figure 5B. in main manuscript<br><br>(Nambiar et al., 2021)	Oral candidiasis (+)	0.43	0.61
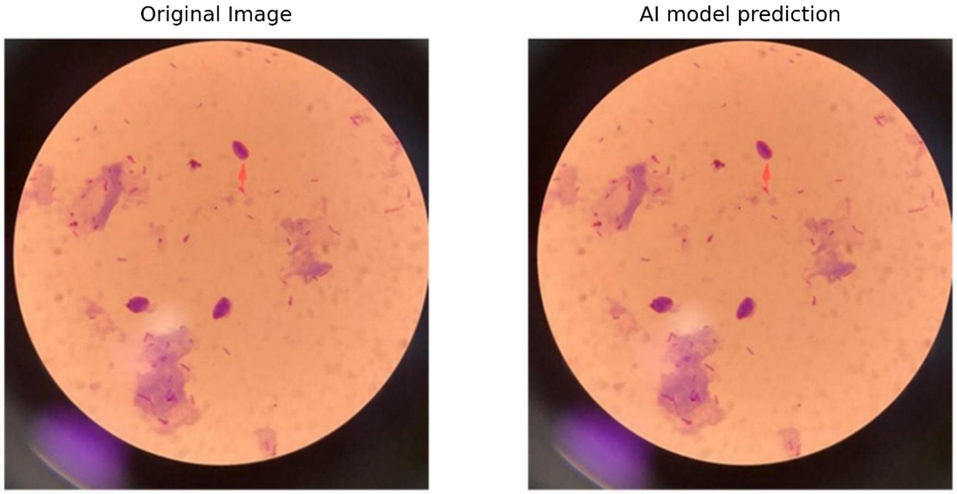<br>Figure 5C. in main manuscript.<br>(Beder et al., 2025)	TV (-)		
https://www.reddit.com/r/microbiology/comments/fijveh/vaginal_discharge_with_intracellular_diplococci/	intracellular diplococci		

	(Gonococci) (-)		
**Average**		0.44	0.6

[a] Model prediction visualizations are provided only for images licensed under CC BY 4.0 or public domain.

References

BEDER, D., ESENKAYA TAŞBENT, F., KıLıÇ HAMZAOĞLU, F., TÜREN DEMIR, E., ÖZDEMIR, M. & ARSLAN, G. K. 2025. Investigation of causative agents of vaginitis in symptomatic and asymptomatic women in Konya, Turkey. *Parasitologia,* 5**,** 15.

CHUDZICKA-STRUGAŁA, I., GOŁĘBIEWSKA, I., BANASZEWSKA, B., TRZCIŃSKI, M., BRUDECKI, G., ELAMIN, W. & ZWOŹDZIAK, B. 2024. Bacterial Vaginosis (BV) and vaginal microbiome disorders in women suffering from Polycystic Ovary Syndrome (PCOS). *Diagnostics (Basel),* 14.

MCGARRY, B. J. 2010. Vaginal Discharge. *Primary Care Procedures in Women's Health.* Springer.

NAMBIAR, M., VARMA, S. R., JABER, M., SREELATHA, S. V., THOMAS, B. & NAIR, A. S. 2021. Mycotic infections - mucormycosis and oral candidiasis associated with Covid-19: a significant and challenging association. *J Oral Microbiol,* 13**,** 1967699.

RAMSAY, S., ASTILL, N., SHANKLAND, G. & WINTER, A. 2009. Practical management of recurrent vulvovaginal candidiasis. *Trends in Urology, Gynaecology & Sexual Health,* 14**,** 18-22.
